# Human health risk from groundwater consumption: A case study in the Apaaso-Tafo Community in the Bono East Region of Ghana

**DOI:** 10.1016/j.heliyon.2025.e42460

**Published:** 2025-02-04

**Authors:** Emmanuel Okyere, Ethel Bentil, Nana Adwoa Kusi-Addai, Lawson Mensah

**Affiliations:** Department of Environmental Science, College of Science, Kwame Nkrumah University of Science and Technology, Kumasi, Ghana

**Keywords:** Groundwater quality, Borehole, Human infection risk, Kintampo, Ghana

## Abstract

Water is a nutrient for life and a resource for human development, and therefore towns and cities often build treatment systems to provide wholesome water for consumption, bathing, washing, etc. For rural communities where centralised water treatment plants are lacking, groundwater (GW) is often the resource of choice due to its relatively low pathogenic loading and turbidity. However, GW is highly susceptible to contamination from anthropogenic activities. In the Bono East region of Ghana, 94 % of solid waste and 89 % of wastewater are disposed of on the roadside without treatment, posing a contamination risk to water resources. Despite the potential for GW contamination in the region, few researchers have assessed GW quality, and none has evaluated the human infectious risk from GW consumption. In this case study, GW quality in the Apaaso-Tafo community in the region was assessed and the infectious health risks posed to consumers were evaluated. Twenty GW samples were collected from ten boreholes. The concentrations of metals (Ca^2+^, Mg^2+^ and Fe) were determined using atomic absorption spectroscopy, bacteria (total and faecal coliform) using Multiple Tube Fermentation and Membrane Filter Techniques, and physicochemical qualities using electrometric, titrimetric, argentometric and spectrophotometric methods. The water quality indices (WQI) and the infection probability from drinking the GW were computed. The WQI showed that most (80 %) GW samples are within acceptable limits. Except for the pH, temperature, turbidity and total coliform bacteria, all parameters met the WHO standards. 40 % of the boreholes contained 50 to 500 cfu/100 mL of coliform bacteria and posed daily infection risks between 1 and 7.63 %. It is recommended that standpipes on boreholes be fitted with membrane or nano filters capable of removing bacteria and viruses to reduce infection risk. The authorities should also monitor GW quality regularly and respond to changes in quality with the appropriate intervention such as issuing ‘boil-before-drinking’ notices to consumers or encouraging parents to provide bottled and sachet water for their young children. Future research with an increased number of communities, sample size and sampling frequency should also be conducted to enhance the generalisability of these findings.

## Highlights

• Most (80%) of groundwater samples in the Apaaso-Tafo Community in Bono East Region of Ghana are within acceptable limits.
• Consumers of contaminated groundwater faces 97–100 % probability of pathogenic infection annually.
• Most groundwater sources in the Apaaso-Tafo community has pH below the 6.5 WHO lower limit but the mean pH of the area is 7.03.
• Mean groundwater temperature in the Apaaso-Tafo community was 27 °C, higher than the recommended 25 °C for drinking water.

## 1 Introduction

Water is vital to both human life and the health of ecosystems. As such, the sixth Sustainable Development Goal (SDG 6) by the United Nations [1] urges that all governments and institutions must strive to protect and improve the availability and quality of water for all citizens [1]. This is because unwholesome water consumption, sanitation and hygiene cause approximately 830,000 deaths and 50 million disability-adjusted life years globally [2], with nearly 36 % of deaths occurring in children under five years old. Unfortunately, even in the most pristine environments with zero anthropogenic interference, water may become contaminated as it passes through the hydrologic cycle; and the level of contamination is exacerbated where human habitation and activities abound [3]. For instance, surface and groundwater resources may be contaminated by leaks from subterranean sewage pipes, abandoned wells, quarries, mines, leachate from landfill sites, use of pesticides and fertilisers in agriculture, chemical spillages, and municipal or industrial wastes [[4], [5], [6]]. Hence, it is prudent to treat water before consumption.

Albeit the body of research that demonstrates the enormous disease burden associated with the consumption of untreated water, many communities in developing nations such as Ghana where access to treated water is lacking have resorted to untreated groundwater for domestic use [7]. Globally, groundwater constitutes fifty per cent of water used in domestic settings including drinking, especially for rural communities with limited water supply infrastructure [1]. In Africa, an estimated 75 % of the population relies on groundwater for household activities [8,9]. In Ghana, about 41 % of households use groundwater, with the majority in rural communities [10], particularly in the Northern Region [11]. This significant reliance on groundwater occurs because it is perceived to be of good quality, useable without treatment, and an economical approach to guarantee the reliability of the water source [1]. However, drinking untreated or contaminated groundwater significantly threatens public health [12]. For example, drinking water contaminated with industrial solvents caused morbidity with cancers being highly prevalent [13]. The consumption of poor-quality groundwater is strongly associated with stunted growth, wasting, underweight and other nutritional deficiency symptoms in children [14]; miners consuming contaminated groundwater suffered prolonged enteric fever outbreaks [15], and the drinking of fluoride-contaminated groundwater led to dental fluorosis in northern Ghana [11,16].

The Kintampo South District in the Bono East region of Ghana is one area where inhabitants heavily rely on groundwater. About 30 % of households in the district depend on groundwater (boreholes/pump/tube wells) as their primary source of water supply, with 40 % of rural and 25 % of urban households drinking groundwater [17]. In the Apaaso-Tafo community within the district, households mainly use groundwater as the primary water source. The Bono East region also has the lowest sanitation performance in the country, with approximately 94 % of households lacking the basic ‘soakaway’ system for wastewater disposal, and 89 % of dwellings practising open dumping of solid waste [17]. According to Fida et al. [6], improperly disposed liquid and solid waste are major causes of groundwater contamination. Therefore, the consumption of untreated groundwater for domestic settings (including drinking) in the Kintampo South District where waste management services are also poor, is overly concerning. Even with these concerns of potential contamination of groundwater from inappropriate waste disposal practices in the area, only one study has assessed groundwater quality in the Kintampo South District [18], and none has examined the infectious risk posed by the consumption of groundwater in the district.

Therefore, it is imperative to assess the physicochemical and bacteriological quality of groundwater in the region to determine the risk posed to consumers. This case study aims to measure the wholesomeness of untreated groundwater consumed by the Apaaso-Tafo community in the Kintampo South District. The study further computes the water quality ratings of groundwater sources and the infectious health risks posed to consumers in the district.

## 2 Materials and methods

### 2.1 Description of the study area

**Fig. 1:**
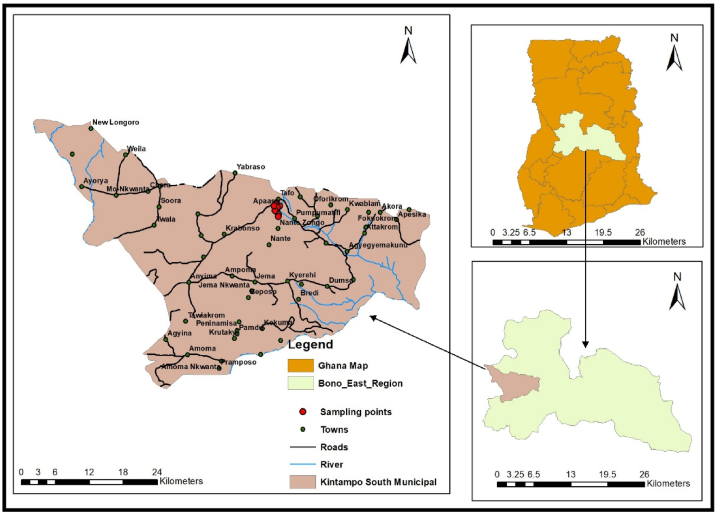
Map of the study area showing sampling points.

The Kintampo South District forms part of the 11 Municipalities and Districts in the Bono East Region of Ghana (Fig. 1). The district is made up of 122 settlements located in the Voltaian basin between latitudes 8°15′ North and 7°45′ South and longitudes 1°20′ West and 2°10′ East, has a population of 101,494 with a female-to-male ratio of 51:49 [19]. The crustal makeup of the area consists predominantly of limestone, shale, mudstone and sandstone. The district experiences annual average rainfall between 1400 and 1800 mm, with a major rainy season starting in early March and a minor season in late August. Monthly temperatures range from 24 to 30 °C, promoting sunny conditions throughout the year (Kintampo Weather Climate, 2021). The climate and vegetation of the geolocation have given rise to beautiful waterfalls and natural forest reserves which are major tourist attractions in the district [20]. Typically, the settlements in the district are farming communities engaged in food crop cultivation and livestock rearing due to the rich soil and vegetation of the land. One of the communities is the Apaaso-Tafo with 1918 people accounting for about 2 % of the district's population [19]. Access to safe water in the district is quite limited as 14 % of the region drinks sachet water and about one-third of the dwellings rely on hand-dug wells and boreholes as drinking and domestic water sources [17].

### 2.2 Sample size and collection

The Apaaso-Tafo community (study area) was initially surveyed to determine the number of functional boreholes, and their locations were marked using the Global Positioning System (GPS). Out of twenty-six borehole standpipes identified, ten (10) were selected using a stratified random sampling method where all boreholes within a 50-m radius are placed in a single stratum, to increase the chances of analysing groundwater from different aquifers or different parts of the same aquifer. Twenty samples were taken from the ten boreholes (Table 1) into 500 mL plastic bottles from their respective standpipes in November 2022. For the physicochemical analyses, the sample was collected into a clean plastic bottle, and for the bacteriological analysis, the sample was collected in a sterile plastic bottle. To achieve a representative sample of the groundwater, the boreholes were purged for about 5 min to remove stagnant water from the pump and water delivery system or until water with stable electrical conductivity and pH flowed from the standpipe. To prevent microbial contamination, ethanol was used aseptically to disinfect the tap and sampling bottle. All the collected samples were clearly labelled and preserved on ice as described by Safo-Adu [21] to keep the samples at about 4 °C before transporting them to the laboratory.

**Table 1:** Identity of boreholes in Apaaso-Tafo community and their locations.

Sample code	Coordinates	Description
BH-1	8.0139N 1.7350W	Malam House
BH-2	8.0209N 1.7326W	Sammy Ahutor House
BH-3	8.0185N 1.7317W	K.K. House
BH-4	8.0062N 1.7327W	Police Barrier 2
BH-5	8.0027N 1.7325W	Burger House
BH-6	8.0194N 1.7303W	Azindow House
BH-7	8.0232N 1.7360W	Nii (New Tafo)
BH-8	8.0175N 1.7339W	WVI 1
BH-9	8.0124N 1.7380W	Apaaso Top 2
BH-10	8.0196N 1.7394W	Gafaru (Masalachi)

### 2.3 Groundwater quality analysis

Physicochemical parameters were measured in situ. pH and temperature were determined using the Hanna HI-98128 pH meter [22]. The electrical conductivity (EC) and total dissolved solids (TDS) were measured using the OHAUS Starter 3100C conductivity meter following the manufacturer's protocol [23]. The turbidity was also analysed using the VELP Scientifica TB1 Turbiditmeter [23]. Before the assessment, the pH meter was calibrated with pH 4 and pH 10 buffer solution whilst the turbidity meter was calibrated with 0.2 and 20 NTU standards solutions. The chloride (Cl⁻), nitrate (NO_3_^−^), ammonia (NH_3_), and total hardness (TH) were analysed at the laboratories of Ghana Water Company Limited in Kumasi. Total hardness, alkalinity and chloride were analysed using electrometric, titrimetric and argentometric techniques [24]. Calcium (Ca^2+^), magnesium (Mg^2+^) and total iron concentrations were determined using flame atomic absorption spectrometer (AAS) fitted with hollow cathode lamps [25]. Ultraviolet–visible spectrophotometry was used for phosphate (PO_4_^3--^) determination. The samples were also analysed for total and faecal (*E. coli*) coliforms using the Multiple Tube Fermentation and Membrane Filter Techniques [26]. The parameters analysed were compared with WHO drinking water standards [27].

### 2.4 Data analysis

Linear regression analysis of the physicochemical parameters was conducted to identify correlations between variables with coefficient (R^2^) greater than 0.5. Principal component analysis was also performed [28] to compare the boreholes and determine the most influential variables that drive the similarities and differences in the groundwater sources.

#### 2.4.1 Water quality index

The Water Quality Index (WQI) is a tool that combines all the essential water quality parameters into a single value [29] for easy communication of the quality of water to all stakeholders [30]. The weighted arithmetic index approach was employed to assess the appropriateness of the groundwater for domestic use. Physicochemical parameters for determining the WQI include temperature, turbidity, TDS, pH, electrical conductivity, total hardness (TH), alkalinity, calcium and magnesium ions, chloride, nitrates and phosphates. The weighted arithmetic water quality index approach categorises the water quality according to its purity (Table 2) using the most frequently measured water quality parameters [31]. Equation (1) was used to calculate the WQI [32,33].

Equation 1 $$WQI=\frac{\sum QnWn}{\sum Wn}$$

**Table 2:** Weighted arithmetic water quality index rating.

WQI Value	Rating of Water Quality	Grading
0–25	Excellent water quality	A
26–50	Good water quality	B
51–75	Poor water quality	C
76–100	Very Poor water quality	D
Above 100	Unsuitable for drinking purposes	E

[32,33].

*Qn* is the quality rating scale for each parameter for the nth water quality parameter and it is calculated using the expression in equation (2), and *W*_*n*_ is the unit weight for each water quality parameter.

Equation 2 $$Qn=\frac{V_{n}-V_{0}}{S_{n}-V_{0}}\times 100$$

*V*_*n*_ is the observed value (estimated concentration/value of the nth parameter in the analysed water, *V*_*o*_ is the ideal value of the nth parameter in pure water which is zero for all parameters except for pH which is 7.0 and DO concentration which is 14.6 mg/L; whilst *S*_*n*_ is the recommended standard value of the nth parameter by WHO [27]. The unit weight, (*W*_*n*_) is inversely proportional to the value of the recommended standards for each water quality parameter and it is given by equation (3), and the constant of proportionality (*K*) is given by equation (4).

Equation 3 $$W_{n}=\frac{K}{S_{n}}$$

Equation 4 $$K=\frac{1}{\sum \left(1/S_{n}\right)}$$

The water analysed is rated as excellent, good, poor, very poor and unsuitable for drinking based on the WQI values shown in Table 2.

#### 2.4.2 Quantitative microbial risk assessment (QMRA)

This study calculated the microbial risks associated with various groundwater sources using four implementation steps: hazard identification, exposure assessment, dose-response, and risk characterisation [34]. The hazard identification is the total coliform bacteria, a group of related bacteria that are not necessarily harmful but indicate the potential presence of pathogenic bacteria such as *E. coli*. The exposure assessment, denoted as dose per day (*D*) is the product of the concentration of total coliform and the volume of water consumed (*V*_*consumed*_) and is expressed as equation (5). The recommended volume of water for direct consumption in tropical settings is 2 L [35,36], but this varies for different people due to lifestyle and climatic factors.

Equation 5 $$D={Conc}_{Total\;coliform\;}\times V_{consumed}$$

The dose-response assessment was conducted using equation (6) based on the Beta-Poisson model [37].

Equation 6 $$P_{infection,day}=1-{\left(1+\frac{D}{N_{50}}\left(2^{\frac{1}{\alpha }}-1\right)\right)}^{-\alpha }$$

Where P_infection, day_ is the probability of an infection per day, N_50_ is the number of pathogens that will infect 50 % of the population exposed and the values for α and N_50_ are 0.135 and 2.11 × 10^6^ respectively. The risk characterisation was performed using equation (7), where 365 days a year was used as the exposure frequency because it is safe to assume that the consumption of water is a daily habit [38].

Equation 7 $$P_{infection,year}=1-{\left(1-P_{infection,day}\right)}^{365}$$

### 2.5 Limitations

This case study was conducted by taking twenty samples from ten boreholes in one out of the 122 communities in the Bono East region, and therefore generalisations of the results should be done cautiously or avoided. The lack of trace metals analyses on the groundwater samples also limits the application of the results in assessing the suitability of groundwater for specialised uses.

## 3 Results and discussion

### 3.1 Physicochemical characteristic of groundwater in Apaaso-Tafo Community

**Fig. 2:**
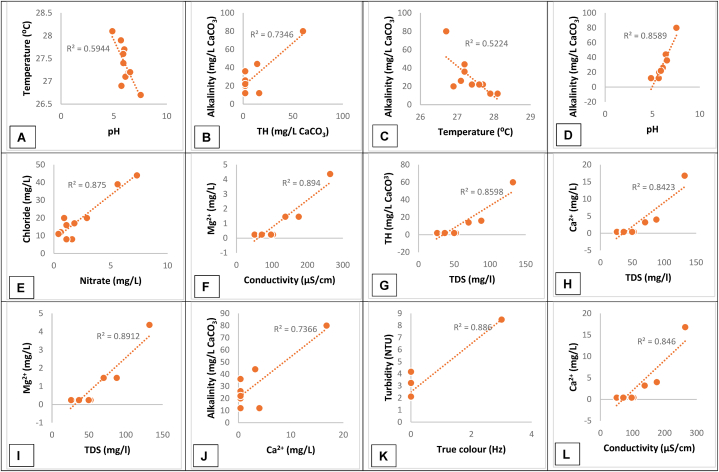
Correlation analysis of groundwater physicochemical properties with R^2^ greater than 0.5 between [A[ temperature and pH, [B] alkalinity and TH, [C] alkalinity and temperature, [D] alkalinity and pH, [E] chlorides and nitrate, [F] magnesium and conductivity, [G] TH and TDS, [H] calcium and TDS, [I] magnesium and TDS, [J] alkalinity and calcium, [K] turbidity and true colour and [L] Calcium and conductivity.

Approximately 25 % of the physicochemical parameters of the groundwater in the Apaaso-Tafo community in the Bono East region of Ghana breached the WHO [27] limits (Table 3). All the groundwater samples had temperatures above the 25 °C WHO [27] threshold for drinking water. The temperatures ranged between 26.7 °C and 28.1 °C and are akin to other findings [39] but higher than with other studies (Table 4). The elevated water temperature may have been influenced by the dry season during which the samples were collected. Warmer water temperature promotes the growth of microbes and negatively impacts the water's taste, odour, colour, and corrosiveness [40]. For BH-10, the turbidity and TSS exceeded the 5 NTU and 0 mg/L limits respectively (Table 3). The pHs of all boreholes apart from BH-1 and BH-10 were below the 6.5 lower limits for potable water (Table 3) although the mean pH is 7.03 (Table 3) and comparable to the literature (Table 4). The predominantly moderately acidic nature of the groundwater in the Apaaso-Tafo district (in the Bono East region) is similar to those found in the neighbouring Northern Region [39], Ejisu Juabeng Municipality of Ashanti Region [41] and Bogoso in the Western Region [42]. The comparable groundwater characteristics in these regions suggest that the bedrock which holds these aquifers may be identical. In contrast, groundwater pH in southern Ghana typically ranges from pH 6.2 to 7.8 [16]. The low pH of the groundwater in the study area will have a corrosive effect on water storage and distribution systems, dissolve plumbing components into drinking water [43], and cause a metallic taste in drinking water from the formation of soluble metal complexes [44], cause stains in laundry, sinks and bathtubs [45], and lead to some individuals experiencing itchy skin when bathing [46]. Therefore, boreholes in the area must be treated by dosing an appropriate concentration of soda ash or sodium hydroxide for pH-correction. The groundwater alkalinity found in this study averaged 26.4 mg/L CaCO_3_, ranged from 12 to 80 mg/L CaCO_3_, correlated with the pH of the samples (Fig. 2, panel A), were within WHO [27] limits and comparable to other studies in Table 4 [41,47]. Since alkalinity is the neutralising ability of water to acids [48] and is primarily caused by the presence of dissolved carbon dioxide species such as bicarbonate, carbonate and hydroxides in groundwater [49], the low alkalinity in all samples except BH-1 and BH-10 resulted in those samples been mildly acidic. All the groundwater samples were categorised as soft water with a total hardness of less than 75 mg/L as CaCO_3_ [50]. The concentrations of calcium (0.4–16.8 mg/L) and magnesium (0.24–4.37 mg/L) ions, ammonia (0 mg/L), chlorides (8–44 mg/L), phosphates (0.08–0.82 mg/L) and alkalinity (12–80 mg/L CaCO_3_) were below their respective WHO [27] limits for drinking water (Table 3). Although nitrates in all samples were below the 10 mg/L limit for the prevention of infant methemoglobinemia [51], recent studies have shown that excessive ingestion of nitrates even at a concentration below the drinking water limit is strongly associated with colorectal and bladder cancer, as well as neural and birth defects [52]. Therefore, daily groundwater consumption from BH-2 and BH-3 raises serious health concerns. The nitrate concentrations found in the Apaaso-Tafo community are equal to levels in the Sokoban area in the Kumasi Metropolis [53], significantly higher than concentrations in the Nanton district in the Northern region [54] and about eighty-time higher than found in Tarkwa in the Western Region [47] as depicted in Table 4. The minimum and maximum electrical conductivity values were 50.4 and 264 μS/cm respectively. Thus, the EC of all samples was below the WHO [27] maximum permissible limit of 400 μS/cm [41].

**Table 3:** Physicochemical and biological parameters of the groundwater samples.

Parameters	Boreholes sampled for groundwater analysis										Descriptive statistics				WHO limits
	BH-1	BH-2	BH-3	BH-4	BH-5	BH-6	BH-7	BH-8	BH-9	BH-10	Min	Max	Mean	STDev	
pH	7.53	5.66	4.85	5.98	5.88	6.40	6.09	5.71	5.89	6.53	4.85	7.53	7.03	0.7	6.5–8.5
True colour (Hz)	0	0	0	0	0	0	0	0	0	3	0	3	0.3	0.9	15.0
Turbidity (NTU)	3.24	2.23	4.17	2.08	2.12	2.27	2.18	2.17	2.11	8.49	2.08	8.49	3.106	1.9	5.00
Conductivity (μS/cm)	264.0	102.7	175.5	50.4	50.9	137.7	70.5	69.4	71.2	97.3	50.4	264	108.96	63.8	400.0
TDS (mg/l)	132.0	52.2	87.7	25.2	25.9	70.0	36.0	35.0	36.6	49.7	25.2	132	55.03	31.8	1000.0
TSS (mg/L)	0	0	0	0	0	0	0	0	0	1	0	1	0	0.3	0
Temperature (⁰C)	26.7	27.9	28.1	27.7	27.6	27.2	27.1	26.9	27.4	27.2	26.7	28.1	27.38	0.4	25.0
TH (mg/L CaCO_3_)	60.0	2.0	16.0	2.0	2.0	14.0	2.0	2.0	2.0	2.0	2	60	10.4	17.3	500
Ca^2+^ (mg/L)	16.8	0.4	4.00	0.40	0.40	3.20	0.40	0.40	0.40	0.40	0.4	16.8	2.68	4.9	100
Mg^2+^ (mg/L)	4.37	0.24	1.46	0.24	0.24	1.46	0.24	0.24	0.24	0.24	0.24	4.37	0.897	1.3	50
Nitrate (mg/L)	1.60	5.60	7.30	1.10	0.60	1.8	0.40	2.90	0.90	1.10	0.4	7.3	2.33	2.2	10
Ammonia (mg/L)	0.00	0.00	0.00	0.00	0.00	0.00	0.00	0.00	0.00	0.00	0	0	0	0.0	1.5
Chloride (mg/L)	8.00	39.0	44.0	16.0	12.0	17.0	11.0	20.0	20.0	8.0	8	44	19.5	11.8	250
Phosphate (mg/l)	0.31	0.08	0.11	0.28	0.12	0.82	0.23	0.09	0.33	0.27	0.08	0.82	0.264	0.2	5.0
Total iron (mg/l)	BDL	BDL	BDL	BDL	BDL	BDL	BDL	BDL	BDL	BDL	0	0	–		0.3
Alkalinity (mg/L as CaCO_3_)	80.0	12.0	12.0	22.0	22.0	44.0	26.0	20.0	22.0	36	12	80	29.6	19.2	100.0
**WQI**	45.0	39.3	51.0	33.3	31.7	39.6	32.7	34.6	33.3	70.7					
**WQ Descriptor**	Good	Good	Poor	Good	Good	Good	Good	Good	Good	Very poor					
**WQ Rating**	B	B	C	B	B	B	B	B	B	D					

Red values breach the WHO [27] drinking water standard; WQI is the water quality index; Min = minimum; Max = maximum; STDev = Standard deviation

**Table 4:** Groundwater quality in different districts and regions in Ghana.

Parameters	This study		[53]		[47]		[54]	
*Study Area* *Region in Ghana* *Descriptive statistics*	Kintampo South District Bono East Region		Kumasi Metropolitan District Ashanti Region		Tarkwa-Nsuaem Municipal Western Region		Nanton District Northern Region	
	Mean	Std	Mean	Std	Mean	Std	Mean	Std
*pH*	7.03	0.7	5.46	0.68	6.8	0.7	7.51	0.12
*Colour*	0.3	0.9	NA	NA	NA	NA	3.75	0.85
*Turbidity (NTU)*	3.11	1.9	3.88	2.53	24.4	28.5	3.61	1.62
*Conductivity (μS/cm)*	109.0	63.8	177.5	180	398.5	292	782.3	91.73
*TDS (mg/l)*	55.03	31.8	133.8	127	271.7	208.2	469.4	55
*TSS (mg/L)*	0	0.3	NA	NA	NA	NA	2.70	1.06
*Temperature (⁰C)*	27.38	0.4	24.6	0.27	NA	NA	23.55	0.31
*Total Hardness (mg/L)*	10.4	17.3	51.1	29.7	323.1	380.6	108.4	6.33
*Ca*^*2+*^*(mg/L)*	2.68	4.9	29.6	17.9	71.8	84.1	24.67	1.82
*Mg*^*2+*^*(mg/L)*	0.897	1.3	21.5	12.3	34.9	48.7	11.36	1.05
*Nitrate (mg/L)*	2.33	2.2	2.64	2.10	178.2	172.5	0.56	0.13
*Ammonia (mg/L)*	0	0	NA	NA	NA	NA	0	0
*Chloride (mg/L)*	19.5	11.8	NA	NA	39.1	23.6	66.71	20.6
*Phosphate (mg/L)*	0.264	0.2	0.178	0.15	13.3	12.2	0.17	0.04
*Alkalinity (mg/L)*	29.6	19.2	32.2	25.6	109.5	92.4	226.9	18.43
*Total coliform (cfu/*100 mL*)*	72	1.468 × 10^2^	8.9 × 10^6^	5.552 × 10^6^	NA	NA	72.07	86.95
*E. coli (cfu/*100 mL*)*	0	0	2.3 × 10^5^	0	NA	NA	53.20	72.54

∗Std = standard deviation; TDS = total dissolved solids; TSS = total suspended solids; NA = not assessed in the study

### 3.2 Water quality index (WQI) of the boreholes in Apaaso-Tafo district

The WQI datasets revealed that 80 % of the groundwater samples were classified as good quality (Table 3) making these findings comparable to results from the Ashanti, Central and Upper East Regions of Ghana [55]. The WQIs of the remaining groundwater samples (BH-3 and BH-10) were 51 and 71, hence rated as poor and very poor water quality due to their high concentration of nitrates and elevated turbidity respectively (Table 3). The quality of groundwater from BH-3 and BH-10 will have serious health implications for the consumers in the Apaaso-Tafo community in the District of Kintampo South, by placing them at a higher risk of water-borne diseases and colorectal and bladder cancer [52]. No sample was classified as unsuitable for drinking (WQI >100), which is similar to reports in Nigeria [56] and affirms the observed wholesomeness of most groundwater sources in West Africa [1,57]. However, the results contrast with the findings elsewhere in Ghana where 7 % of samples [55] and in India [32] where all samples were categorised as unsuitable for consumption.

### 3.3 Correlations between physicochemical parameters of the groundwater

Linear regression analysis of the physicochemical parameters revealed several correlations with moderate to strong coefficient values (R^2^ > 0.5) between twelve water quality indicators (Fig. 2, panels A–L). TDS correlated positively with TH, Mg^2+^ and Ca^2+^ (Fig. 2, panels G, H and I). Turbidity highly correlated with true colour (Fig. 2, panel K) at R^2^ of 0.89 whilst chloride and nitrate concentrations correlated at R^2^ of 0.88. The alkalinity of the groundwater also correlated with Ca^2+^, total hardness and pH at R^2^ > 0.7 (Fig. 2, panels B, D and J).

### 3.4 Principal component analysis of groundwater characteristics

Using the Kaiser [58] criterion, principal components (PCs 1, 2, and 3) with eigenvalues greater than unity were retained. The three PCs cumulatively accounted for 91.14 % of the total explainable variance, as observed in Table 5. PC1 (46.81 %), which accounts for almost half the total explanatory variance, is primarily driven by the weighted sum of water hardness and salinity variables, such as alkalinity, total hardness, calcium, magnesium, TDS, and EC. The eigenvectors in PC2 (26.32 %) were governed by the weighted difference of Cl^−^ (4.19), TSS (−4.19), and True colour (−4.19), highlighting the influence of ionic content. Conversely, PC3 (18.01 %) captures variations associated with physical water properties like turbidity, true colour, and total suspended solids, as indicated in Table 5.

**Table 5:** Principal components and the variable loading on PCs.

PC	Eigenvalue	% Variance	Cumulative	Variables	PC 1	PC 2	PC 3
					46.81 %	26.32 %	18.01 %
1	6.55	46.81 %	46.81 %	pH	5.75	−2.40	−0.74
2	3.68	26.32 %	73.13 %	True color	0.26	**−4.19**	**2.26**
3	2.52	18.01 %	91.14 %	Turbidity	1.12	−2.98	**2.89**
4	0.81	5.82 %	96.96 %	EC	5.86	2.18	1.18
5	0.29	2.09 %	99.05 %	TDS	5.87	2.15	1.19
6	0.11	0.75 %	99.80 %	TSS	0.26	**−4.19**	**2.26**
7	0.02	0.13 %	99.93 %	Temp	−4.42	2.49	1.25
8	0.01	0.06 %	99.99 %	TH	**6.51**	1.64	0.21
9	0.001	0.01 %	100.00 %	Ca^2+^	6.50	1.58	0.19
10	9.66E-32	0.00 %	100.00 %	Mg^2+^	6.48	1.78	0.24
				NO_3_^2-^	−1.32	4.05	2.13
				NH_3_	0.00	0.00	0.00
				Cl^−^	−2.95	**4.19**	1.50
				PO_4_^3-^	2.71	−1.15	−1.05

**Fig. 3:**
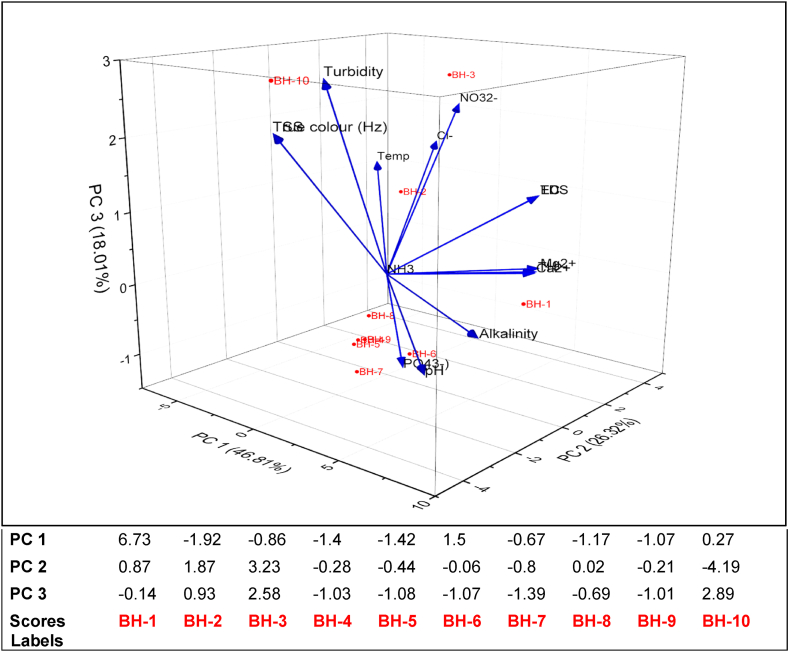
Biplot of the PCs from the characteristics of the boreholes sampled.

The biplot (Fig. 3) indicates how the BHs align/cluster with the 3 PCs and which variables drive the clustering. BH-1 and BH-6 are positively aligned with PC1, suggesting a close association with water hardness and salinity parameters like alkalinity, TDS, and TH. BH-2 and BH-3 on the other hand, exhibit high positive scores on PC2, highlighting their strong correlation with ionic content (e.g., NO_3_^2−^ and Cl^−^), with BH-3 also showing some association with physical water quality variables in PC3. In contrast, BH-4, BH-5, and BH-7 to BH-9 have predominantly negative scores across PCs (Table 5), suggesting weak contributions from the driving variables. BH-10 stands out as an outlier with a highly negative score on PC2 (ionic content) but a strong positive influence from PC3, driven by turbidity and physical water properties. The physicochemical characteristics of BH-1 stand out from the rest (Fig. 3), having the highest pH of 7.53, TDS of 132 mg/L, electrical conductivity of 264 μS/cm, total hardness of 60 mg/L as CaCO_3_, calcium ion concentration of 16.8 mg/L and alkalinity of 80 mg/L as CaCO_3_. These characteristics suggest that the geology of the area of BH-1 may be significantly different. Thus, the rock formation may contain deposits of calcite (calcium carbonate crystals) which has dissolved into the groundwater by the infiltration of acidic rainwater which recharges the aquifer in that area, resulting in relatively higher alkalinity, elevated calcium and carbonate ions, total hardness and the corresponding slightly alkaline pH (Fig. 3), a phenomenon also found in the Dahomeyan and the Togo Structural units in southern Ghana [59]. Groundwater resources in Ghana are mostly contained in basement complexes or consolidated rock formations. These are usually metamorphic and crystalline igneous rocks located in the west, south, central and north basins and cover 54 % of the country [57].

### 3.5 Infectious risk from groundwater consumption in Apaaso- Tafo district

#### 3.5.1 Microbial contamination

None of the boreholes analysed contained faecal coliform but four out of the ten boreholes exceeded the minimum contaminant level of zero cfu/100 mL for total coliforms as recommended by WHO [27]. 40 % of the samples, boreholes BH-1, BH-2, BH-7 and BH-10, had total coliform concentrations of 100, 500, 70 and 50 cfu/100 mL respectively (Table 6) which signifies the occurrence of microbial contamination [53]. The presence of coliform bacteria in the groundwater may originate from latrine pits, seepage from septic tanks [60] or inappropriate disposal of solid waste and wastewater in the area. The coliform bacteria make the groundwater unfit for drinking unless it is boiled or disinfected with hypochlorite, ozone or other oxidising agents [61]. The levels of coliform bacteria found in the aforementioned boreholes increase the likelihood for other pathogenic coliforms to be present in the groundwater which could result in typhoid fever, gastroenteritis and diarrhoea with dysentery [62]. It is common to find groundwater resources to contain such levels of total coliform bacteria [54,63,64], as shown in other districts in Ghana (Table 4). Hence prior treatment of groundwater is necessary to protect the health of the community it serves.

**Table 6:** Bacteriological concentrations and infection risk of groundwater in the Apaaso-Tafo community in Bono East Region of Ghana.

Sample	Faecal coliform (cfu/100 mL)	Total Coliform (cfu/100 mL)	Dose (cfu)	P_infection,_ daily	P_infection,_ year	P_infection,_ daily (%)	P_infection,_ year (%)
BH-1	0	100	2000	0.0198	1	1.98	99.93
BH-2	0	500	10000	0.0763	1	7.63	100
BH-3	0	0	–	–	–	–	–
BH-4	0	0	–	–	–	–	–
BH-5	0	0	–	–	–	–	–
BH-6	0	0	–	–	–	–	–
BH-7	0	70	1400	0.0142	0.9946	1.42	99.46
BH-8	0	0	–	–	–	–	–
BH-9	0	0	–	–	–	–	–
BH-10	0	50	1000	0.01	0.9745	1	97.45
WHO limits	0	3	–	–	–	–	–

#### 3.5.2 Quantitative microbial risk assessment

Based on an average water consumption rate of 2000 mL/d [35,36], an individual drinking water from BH-1 will ingest 2 × 10^3^ coliform bacteria each day, which poses a daily infection risk of 1.98 % and an annual infection risk of 99.93 % (Table 6). BH-2 poses the highest daily risk of 7.63 % with a yearly infection risk of 100 %. BH-7 and BH-10 present consumers with 97.5 % and 99.5 % probability of yearly infection (Table 6). In contrast with farmers using contaminated irrigable water [65] and communities in the Tano North Municipality of Ghana [66], the Apaaso-Tafo community is at a significantly high risk of infection. The risk uncovered is comparable to findings elsewhere [37] but exceeded the WHO [27] limit of 10^−4^. This infectious risk will place a disease burden on the population [2] and impact the development of children [14]. To reduce the infectious probability in the community from groundwater consumption, point-of-use water treatment systems such as nanofiltration and membrane filtration technologies which have been proven to effectively remove bacteria and viruses from water [67] and save costs should be deployed. The Kintampo South District Authority should regularly monitor groundwater quality in the area, at least monthly in the dry season and weekly in the rainy season, and advise the public periodically when changes in water quality are observed.

## 4 Conclusion and recommendation

20 % of groundwater consumed in the Apaaso-Tafo community of Kintampo South District is unsuitable for drinking due to high turbidity, low pH or elevated temperatures above the WHO [27] standard for safe drinking water. The mean pH of groundwater in the area is neutral (7.03) although 80 % of samples had acidic pH below the WHO [27] limits. This suggests that boreholes in the area would benefit from a portable low-cost point-of-use pH-correction technology or treatment methods such as the installation of acid neutralising filter or injection of sodium hydroxide to reduce the acidic content of the groundwater. The PCA revealed that groundwater quality from the boreholes differed. They clustered either by positive association with water hardness and salinity parameters or by positively aligning with ionic concentrations. Thus, different boreholes will require different point-of-use treatment systems based on the groundwater quality emanating from that area. Bacteriological contamination (total coliforms) was identified in 40 % of the samples with concentrations ranging from 50 to 500 cfu/100 mL, thereby breaching the WHO [27] standards. This posed a daily infection probability of 1–7.6 %, and an annual probability of pathogenic infection of 97–100 %. The coliform contamination and the subsequent infectious risk will significantly impact the health of the Apaaso-Tafo community, especially children under five years old and may contribute to child mortality in the community. The researchers therefore recommend that public borehole standpipes be fitted with nanofiltration or membrane filters with proven efficacy in removing pathogens. Otherwise, parents should be educated and encouraged to purchase bottled and sachet water for their infants or boil the groundwater and allow it to cool in a clean bottle before feeding the water to children. It is also recommended that the Kintampo South District Authority monitors groundwater quality regularly to understand the changing infectious risk throughout the year and educate citizens accordingly. Further studies involving multiple communities and a larger sample size to assess the concentrations of heavy metals along with the physicochemical and bacteriological quality of groundwater in the Bono East region should be conducted to improve the generalisability of the findings and overcome the limitations of this study.

## CRediT authorship contribution statement

**Emmanuel Okyere:** Resources, Investigation. **Ethel Bentil:** Writing – review & editing, Project administration, Formal analysis. **Nana Adwoa Kusi-Addai:** Validation, Data curation. **Lawson Mensah:** Writing – original draft, Supervision, Conceptualization.

## Data availability statement

Data included in the article/supplementary material is referenced in the article.

## Declaration of competing interest

The authors declare that they have no known competing financial interests or personal relationships that could have appeared to influence the work reported in this paper.
